# Looking Beyond the Obvious: Subtle Cortical Metastasis Masked by Gonarthrosis

**DOI:** 10.5334/jbr-btr.1128

**Published:** 2017-02-20

**Authors:** Seema Doering, Cedric Boulet, Sokol Malasi, Maryam Shahabpour, Johan De Mey, Michel De Maeseneer

**Affiliations:** 1 UZ Brussel, BE

**Keywords:** unusual, bone metastases, cortical

An 82-year-old woman with known breast carcinoma presented with complaints of pain in both knee joints, which had worsened on the right side in recent months.

X-rays of both knee joints, including an AP and a lateral view, revealed moderate to severe osteoarthritis, which was initially thought to be the reason for her complaints. However, on closer inspection, a subtle lysis in the medial cortex of the metaphysis of the right femur with discrete subjacent diffuse area of medullary sclerosis was seen (Figure 1). The craniocaudal length of the lesion was 28 mm and the depth of the lesion including the subcortical medullary sclerosis was 14 mm. Neither a periosteal reaction nor an associated soft tissue mass was visible on X-rays.

**Figure 1:**
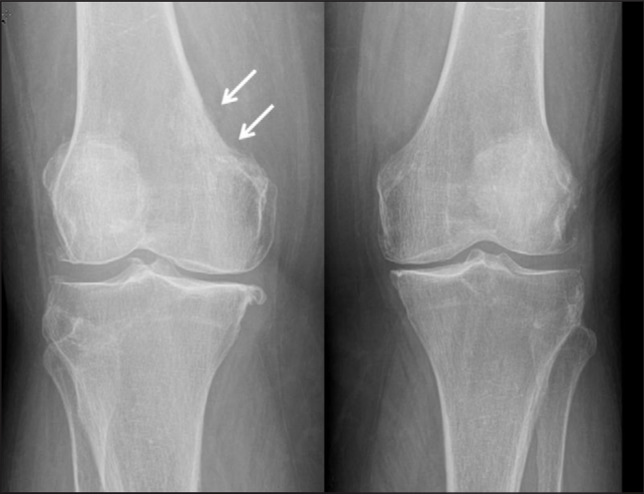
AP views of both knee joints: Obvious bilateral gonarthrosis. The erosive lesion in the medial cortex of distal metaphysis of right femur and associated discrete subcortical sclerosis (arrows) can be easily missed. Compare with the smoothly outlined medial cortex of left femur.

Skeletal scintigraphy confirmed a metabolically active focus in the same location (Figure 2) and revealed multiple other skeletal metastases.

**Figure 2:**
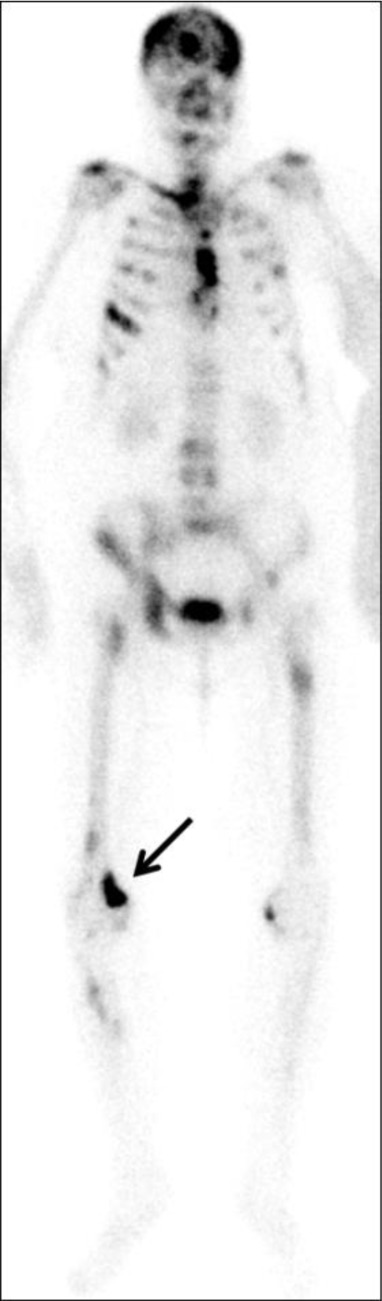
Skeletal scintigraphy – corresponding metabolically active lesion in the right femur (arrow) and multiple other widespread skeletal metastases.

A subsequent CT scan confirmed the presence of an ill-defined cortical metastasis with associated subjacent diffuse medullary sclerosis. It also revealed a discrete associated soft tissue mass (Figure 3A, B and C).

**Figure 3:**
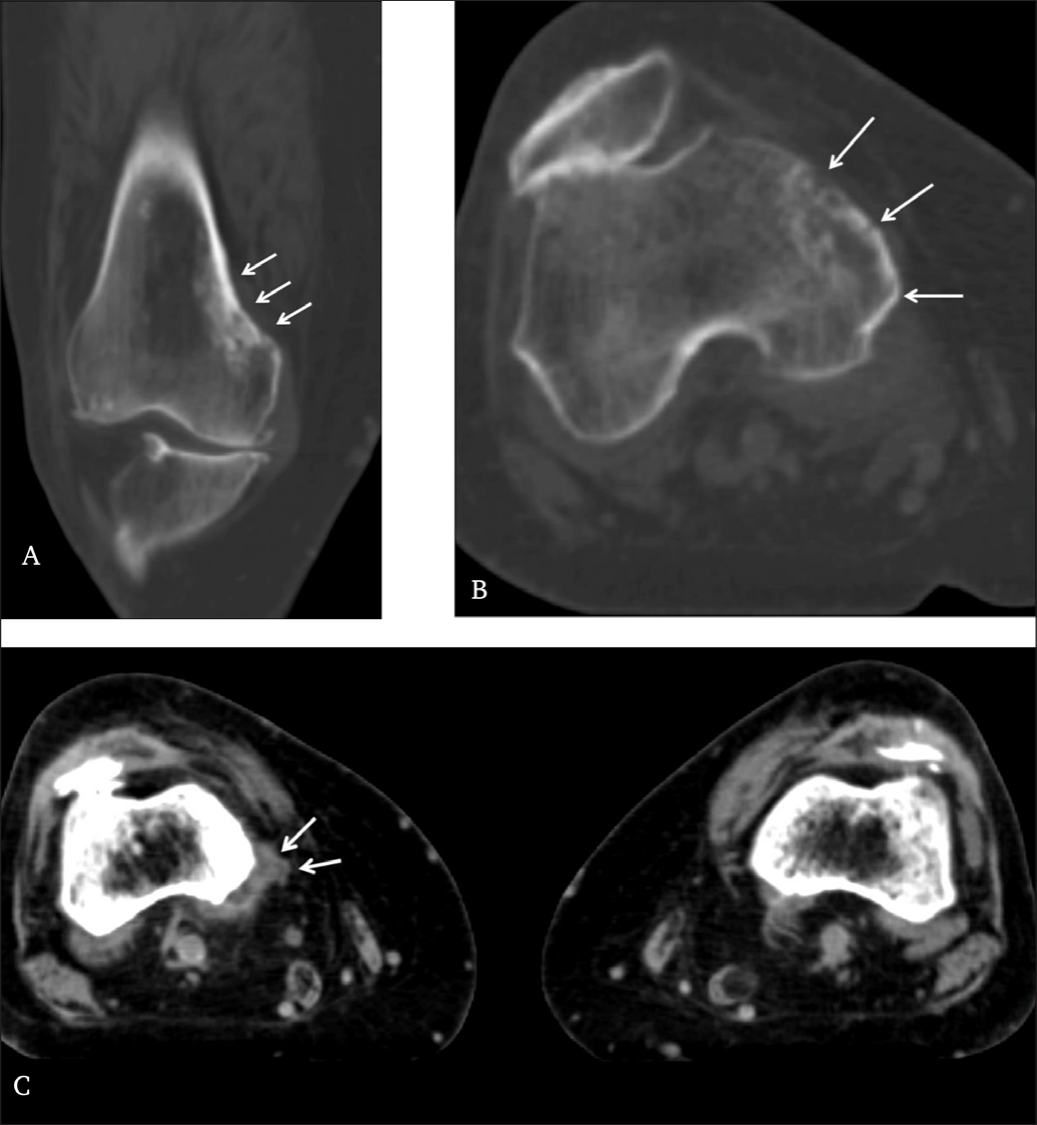
**A.** Coronal reconstructed CT image and **B.** axial CT image through distal right femur in bone window show the extent of the cortical metastases and underlying sclerosis more clearly (arrows). **C.** Axial CT scan through distal femur in soft tissue window shows a discrete associated soft tissue mass posteromedially on the right side, compared with the normal left side (arrows).

## Comment

This lesion could have been easily missed on the initial X-ray of the knee for at least two reasons. First, the presence of obvious osteoarthritis gave an explanation for her knee pain and distracted attention away from the more significant metastatic lesion. This is typically known as ‘satisfaction of search’. Secondly, cortical metastasis is relatively uncommon.

Greenspan and Norman [1] found 22 cortical metastases in 11 patients and classified cortical metastases into four distinctive patterns based on their appearances:

I: small focal lesions of marginal cortical destruction (cookie-bite or cookie-cutter lesions) (7/22).

II: large osteolytic cortical lesions (4/22).

III: saucerized intracortical lesions with well-defined periosteal reaction (5/22).

IV: lesions with predominant cortical destruction extending into the soft tissue and the cancellous bone (6/22).

The lesion in our case report would classify as type IV above.

Coerkamp and Kroon [2], in their study of 26 patients with solitary cortical bone metastases, classified them based on their appearances slightly differently:

Type 1: completely intracortical (10/26).

Type 2: cortical with extension in the soft tissues (7/26).

Type 3: cortical with extension into the medulla (6/26).

Type 4: subperiosteal origin with cortical erosion giving the appearance of a saucer (3/26).

Our lesion is a combination of Type 2 and Type 3 according to this classification.

In our patient, the cortical metastasis occurs in the femur, which is reported to be the commonest location (73–86%) for cortical metastases [1,2,3]. Other locations (15–25%) in the humerus, tibia and ulna have also been described [2,3].

The primary tumor in our case was breast carcinoma. While some authors have found cortical metastases solely in primary from lung carcinoma [1], others mention primary tumors from other organs with variable incidence [2,3]. Renal cell carcinoma as primary tumor has been reported with nearly an equal incidence as lung carcinoma [2,3]. Primary breast tumor was found in one out of 26 cases by Coerkamp et al. [2] and in as many as ten out of 36 cases by Hendrix et al [3]. Much lower incidences of one case each in a series 26 and 36 cortical metastases respectively [2,3] were from other primaries such as neuroblastoma, melanoma, hepatoma, thyroid, pancreas, larynx and uterus.

The pathogenesis of cortical metastasis and its propensity for femur may be related to the unique vascular supply to the cortex of long bones.

Trias and Fery [4] have demonstrated two distinct but freely anastomosing vascular systems within the cortex of femur. The first is in continuity with endosteal and periosteal vascular systems; the second arises from branches of the nutrient artery. One could speculate that cortical metastases either develop from tumor emboli caught in these vascular networks and/or certain tumor cell types have an inherent affinity for the vessels of bone cortex.

In summary, elderly patients with known primary tumors and multiple bone lesions should alert one to the possibility of bone metastases. Unusual lesions should also be considered suspect in such cases. One has to be especially wary of co-existing ‘maskers’ like ostoearthritis in this case.
